# From Eradication to Holistic Regeneration: Pharmaceutics Strategies for Reshaping Gastric Homeostasis Against *H. pylori* Infection

**DOI:** 10.3390/pharmaceutics18030337

**Published:** 2026-03-09

**Authors:** Qingsong Qu, Wanhong Zhu, Xingjian Song, Jingqi Zeng, Jie Lin, Xia Ding

**Affiliations:** 1 School of Traditional Chinese Medicine, Beijing University of Chinese Medicine, Beijing 100029, China; quqingsong@bucm.edu.cn (Q.Q.); 18801357663@163.com (X.S.); 2 Dongzhimen Hospital, Beijing University of Chinese Medicine, Beijing 100029, China; zhuwanhong64@bucm.edu.cn; 3 School of Chinese Materia Medica, Beijing University of Chinese Medicine, Beijing 100029, China; zjingqi@163.com; 4 National Institute of TCM Constitution and Preventive Medicine, Beijing University of Chinese Medicine, Beijing 100029, China; 5 Research Center for Spleen and Stomach Diseases of Traditional Chinese Medicine, Beijing University of Chinese Medicine, Beijing 100029, China

**Keywords:** *Helicobacter pylori*, gastric-targeted delivery, mucosal repair, biofilm, postbiotics

## Abstract

Although the eradication of *Helicobacter pylori* is critical for preventing gastric cancer, current therapies often overlook the restoration of the gastric microenvironment, leading to a prevalence of delayed tissue healing and dysbiosis. Consequently, many patients remain in a persistent pathological state despite successful *H. pylori* clearance, presenting a major bottleneck in clinical treatment. This review summarizes recent advancements in gastric-targeted drug delivery systems, illustrating the evolution from a singular antibacterial approach to an integrated sequential strategy encompassing clearance, repair, and homeostasis reconstruction. We examine smart gastro-retentive and nanodelivery systems designed to overcome physiological barriers, highlighting formulations that extend gastric residence time and maintain local drug concentrations above the Minimum Inhibitory Concentration for prolonged periods. Furthermore, we discuss spatiotemporally controllable biomaterials, such as Janus hydrogels and ROS-responsive carriers. These systems demonstrate distinct pH-dependent release kinetics and high stability in simulated gastric fluids, effectively preserving bioactive payloads to modulate the immune microenvironment. By facilitating the transition from pro-inflammatory to anti-inflammatory phenotypes, these biomaterials support epithelial regeneration. The review concludes with an analysis of postbiotics and the proposed holistic strategy, offering a promising therapeutic framework for mitigating the inflammation-to-cancer transition and promoting gastric health remodeling.

## 1. Introduction

*Helicobacter pylori* (*H. pylori*) infection is a primary pathogenic factor contributing to chronic gastritis, gastric ulcers, and gastric cancer [1,2]. By secreting virulence factors such as cytotoxin-associated gene A (CagA) and vacuolating cytotoxin A (VacA), *H. pylori* induces persistent mucosal inflammation, epithelial damage, and microecological dysbiosis, markedly elevating the risk of malignancy [3,4]. Recent studies have explored advanced delivery systems to improve treatment outcomes for *H. pylori* infections. For instance, a novel mucus and biofilm-penetrating nanoplatform has been developed, which utilizes ultrasound-induced free radicals to target and treat *H. pylori* infections more effectively [5]. Although eradication therapy remains the cornerstone for preventing gastric cancer and ulcer recurrence, clinical management faces substantial challenges [6,7,8], including antibiotic resistance, insufficient drug exposure, rapid gastric emptying [9].

However, *H. pylori* eradication marks only the initial step in restoring gastric health [10]. Gastric mucosal repair is a complex, long-term process involving inflammation resolution, epithelial regeneration, revascularization, and oxidative stress regulation [11]. This reparative process is frequently impeded by a vicious cycle of barrier damage and aggravated inflammation, causing many patients to exhibit persistent pathological states and symptoms, such as pain and bleeding, even after successful *H. pylori* clearance [12,13,14,15]. In this context, new strategies, such as the use of copper-bearing metal–organic frameworks (MOFs), have shown promise in overcoming barriers like mucus penetration. These MOFs not only penetrate mucus but also clear *H. pylori* efficiently from the gastric mucosa, providing a multi-effective solution to persistent infections [16]. Consequently, accelerating the reconstruction of the mucosal barrier to disrupt this malignant cycle has become a critical bottleneck in current therapeutic regimes [17].

Gastric-targeted drug delivery has emerged as a promising strategy to address these challenges [16]. Gastro-retentive drug delivery systems (GRDDS) prolong gastric residence time, enhance local drug concentration, and protect therapeutic agents from degradation by gastric acid or enzymes [18,19]. Currently, this field is evolving from a singular focus on pathogen eradication to an integrated paradigm of “Eradication–Modulation–Regeneration”. Driven by breakthroughs in materials science and nanotechnology, novel carriers such as probiotics, exosomes, and smart hydrogels are being explored for their multifunctional capabilities. Current preclinical studies suggest these platforms could offer anti-inflammatory, antioxidant, and barrier-repairing effects, representing a promising experimental approach to mitigate mucosal damage [20,21].

With the advancement of precision medicine, gastric-targeted delivery is increasingly shifting toward customized treatments based on patient-specific pathological characteristics, such as the severity of inflammation and microbiota profiles [22,23]. Optimizing delivery systems to modulate the local microenvironment, particularly through the restructuring of the gastric microbiota, has the potential to fundamentally enhance treatment outcomes and play a pivotal role in the prevention and interception of gastric cancer [24,25,26].

## 2. Gastric Mucosal Repair After *H. pylori* Eradication

### 2.1. Immune Response Remodeling and the Critical Period for Blocking the Inflammation-to-Cancer Transition

**Figure 1:**
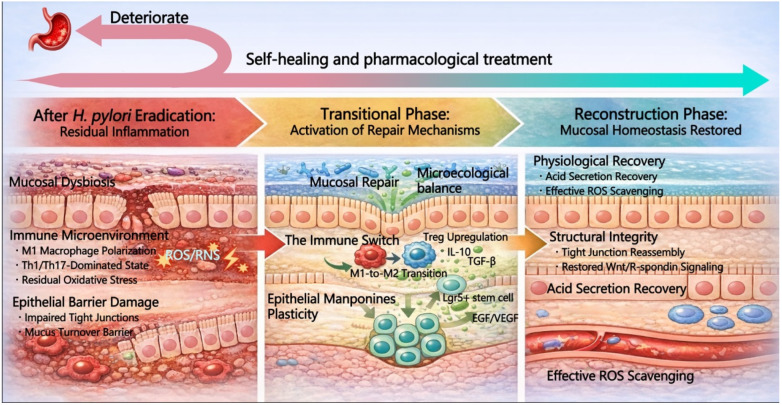
Post-eradication trajectory of gastric mucosal remodeling. Following *H. pylori* eradication, gastric mucosal remodeling may proceed along two divergent trajectories: continued deterioration driven by residual inflammation or progression toward self-healing that can be accelerated by pharmacological intervention and typically evolves through a modulation phase and a reconstruction phase. In the residual-inflammation state, persistent mucosal dysbiosis and a pro-inflammatory immune microenvironment prevail. Specifically, this environment is characterized by M1 macrophage polarization and Th1/Th17-skewed responses. These factors sustain oxidative stress mediated by ROS and RNS, which subsequently aggravates epithelial barrier injury and disrupts mucus turnover. During the modulation phase, repair programs are initiated through an immune switch toward M2 polarization with Treg upregulation and increased IL-10 and TGF-β, together with restoration of microecological balance and enhanced epithelial plasticity, accompanied by regenerative signaling such as Lgr5-positive stem-cell activation and EGF- and VEGF-associated cues. During the reconstruction phase, mucosal homeostasis is re-established through physiological recovery, including acid secretion restoration and improved redox control, and through structural repair, including tight-junction reassembly and reactivation of Wnt and R-spondin signaling, ultimately rebuilding a resilient gastric mucosal barrier.

The immune response induced by *H. pylori* infection undergoes a complex process of spatiotemporal remodeling following eradication therapy. Although eradication removes the pathogen, the restoration of the gastric mucosal immune microenvironment is not instantaneous and depends on a critical period for immune transition. Recent studies have highlighted the role of spatiotemporally controlled biomaterials, such as Janus hydrogels and ROS-responsive carriers, which modulate the immune microenvironment during gastric mucosal repair, aiding in the transition from pro-inflammatory to anti-inflammatory states [27].In the early phase of clearance, a moderate pro-inflammatory response aids in thoroughly eliminating residual pathogens and necrotic tissue [28,29]. However, the immune system must subsequently shift rapidly from a T helper 1 (Th1)/T helper 17 (Th17) dominated defensive pro-inflammatory state to a Treg-dominated reparative anti-inflammatory state [30]. The timing of this immune transition is crucial. If the transition is timely, inflammation resolves rapidly, initiating repair; conversely, a delay may trap the gastric mucosa in chronic inflammation, leading to cumulative damage and the progression of precancerous lesions such as atrophy and intestinal metaplasia [31,32]. Therefore, the upregulation of regulatory T cells (Tregs) and the timely release of anti-inflammatory cytokines, notably interleukin-10 (IL-10) and transforming growth factor-β (TGF-β), serve not only as mechanisms to mitigate cytokine storms but also as molecular hallmarks defining the onset of the repair phase (Figure 1) [32].

In this process, macrophage phenotypic polarization acts as the switch for immune transition. Following pathogen clearance, macrophages must polarize from the pro-inflammatory M1 phenotype to the reparative M2 phenotype [33,34,35,36,37,38,39,40,41]. If this polarization process is impeded and M1 macrophages persist, they continue to damage the mucosal barrier by secreting ROS and inflammatory mediators.

Furthermore, the role of immune cells in gastric mucosal repair is not limited to local effects but involves the synergistic regulation of the systemic immune system [42]. Studies indicate that the immune repair process of the gastric mucosa is often accompanied by the remodeling of intestinal immune responses, where the immune interaction of the gut-stomach axis has a profound impact on maintaining immune homeostasis throughout the digestive system [43,44]. This cross-organ immune crosstalk provides macroscopic immune regulatory support for gastric mucosal repair after *H. pylori* eradication and suggests potential therapeutic strategies to assist gastric immune remodeling by modulating the intestinal microbiota [45]. Therefore, an ideal delivery system is required not only to eradicate bacteria but also to function as an immunomodulator, driving the M1-to-M2 transition at the appropriate biological timing [46].

### 2.2. Epithelial Regeneration and the Regulation of Stem Cell Plasticity

The reconstruction of the gastric mucosal barrier is particularly critical following *H. pylori* eradication [47]. This process is heavily reliant on the mobilization of gastric epithelial stem/progenitor cells and the functional restoration of their associated stem cell niche [48]. During *H. pylori* infection, bacterial virulence factors, such as CagA, not only directly damage epithelial cells but also disrupt signaling homeostasis within the stem cell niche, leading to an imbalance in epithelial proliferation and differentiation [49,50]. Consequently, a core objective following eradication therapy is to rectify this imbalance, gradually restoring the structural integrity and secretory function of gastric glands through the re-equilibration of cell lineages.

In this process, Epidermal Growth Factor (EGF), Vascular Endothelial Growth Factor (VEGF), and the Trefoil Factor family (TFFs) constitute a critical signaling network initiating regeneration. VEGF facilitates microvascular angiogenesis, providing essential oxygen and nutrient support to high-metabolic regeneration zones, which is crucial for mucosal repair within a hypoxic environment. TFFs, particularly trefoil factor 2 (TFF2), not only promote the coverage of damaged surfaces via restitution but also function to stabilize the mucus layer and exert anti-apoptotic effects, thereby providing physical protection for stem cell differentiation [51]. Furthermore, while eliminating residual pathogens, Interferon-gamma (IFN-γ) enhances immune surveillance by upregulating major histocompatibility complex (MHC) molecules; it acts synergistically with growth factors to ensure that regeneration proceeds within a sterile and controlled environment of inflammation resolution [50].

Organoid-based studies have revealed the remarkable plasticity exhibited by gastric epithelial cells during *H. pylori* infection and repair [51,52]. Research confirms that under the stress of inflammatory injury, mature gastric Chief Cells can undergo dedifferentiation, reverting to a progenitor state expressing stem cell markers, such as Troy+, to replenish the compromised stem cell pool [52,53]. Subsequently, following *H. pylori* eradication and the restoration of the Wnt/R-spondin signaling pathway, these cells can re-initiate the “redifferentiation” program to reconstruct functional gastric gland units. This discovery not only elucidates the source of the gastric mucosa’s potent regenerative potential but also suggests that targeted modulation of the “dedifferentiation-redifferentiation” axis may represent a novel strategy for accelerating mucosal repair post-eradication [54].

### 2.3. Microbiota Reconstruction

Following *H. pylori* eradication, the restoration of gastric microbiota and the enhancement of microbial diversity play a pivotal role in gastric mucosal repair. While eradication therapy eradicates the pathogenic *H. pylori*, it may concomitantly perturb other gastric microbial communities, leading to mucosal dysbiosis. Consequently, the recovery and reconstruction of the post-eradication microbiota have emerged as critical steps in maintaining gastric health [45].

Research demonstrates that the diversity of the gastric microbiota gradually recovers after eradication. The colonization of beneficial strains, such as *Lactobacillus* and *Bifidobacterium*, aids in inhibiting pathogen recolonization and mitigating inflammatory responses through interactions with the mucosal immune system [46]. These probiotics maintain gastric microecological balance not only via the competitive exclusion of pathogens but also by generating beneficial metabolites, such as short-chain fatty acids (SCFAs), which further strengthen the gastric mucosal barrier function [47]. By promoting local immunomodulation and antioxidant responses, these bacteria can mitigate gastric mucosal inflammation and accelerate the repair process [48].

### 2.4. Reconstruction of Physiological Barriers

The reconstruction of the physiological barrier following *H. pylori* eradication is a synchronized process involving the reversion of the biochemical microenvironment and the reorganization of physical structures (Figure 1). First, the recovery of gastric acid secretion not only signifies the regeneration of functional parietal cells but, more critically, regulates the local pH environment to inhibit residual oxidative stress (ROS/RNS) persisting from the infection, thereby creating optimal metabolic conditions for epithelial proliferation and repair [55]. This normalization of the acidic milieu further stimulates the activity of antioxidant molecules, like Vitamin C, assisting in the scavenging of free radicals [56].

Concurrently, the core of physical barrier repair lies in the restoration of the integrity of intercellular Tight Junctions (TJs). During infection, the expression of key structural proteins, including Claudin-1, Claudin-4, and Occludin, is suppressed by *H. pylori* virulence factors. Following eradication therapy, the expression levels of these proteins are significantly upregulated, rapidly sealing the compromised intercellular gaps and reducing mucosal permeability to pepsin and carcinogens. Accompanied by the recovery of mucin secretion function, a dense and continuous mucus-tight junction composite barrier is reconstructed, serving as a robust shield against luminal aggressors and maintaining long-term mucosal homeostasis.

## 3. Roles and Challenges of Gastric-Targeted Drug Delivery

A critical challenge in gastric-targeted drug delivery systems lies in prolonging the gastric residence time (GRT) to enhance local drug exposure. Low-density or ultra-porous scaffold materials, such as floating tablets, microsponges, and 3D-printed porous tablets, have been extensively employed in the development of gastro-retentive drug delivery systems (GRDDS). The design of these materials prolongs gastric residence time. For instance, optimized floating tablets have been shown to maintain buoyancy for over 12 h in vitro and prolong gastric retention to 24 h in vivo in beagle dog models, ensuring sustained drug release [57].

### 3.1. Buoyancy and Gastro-Retentive Systems

**Figure 2:**
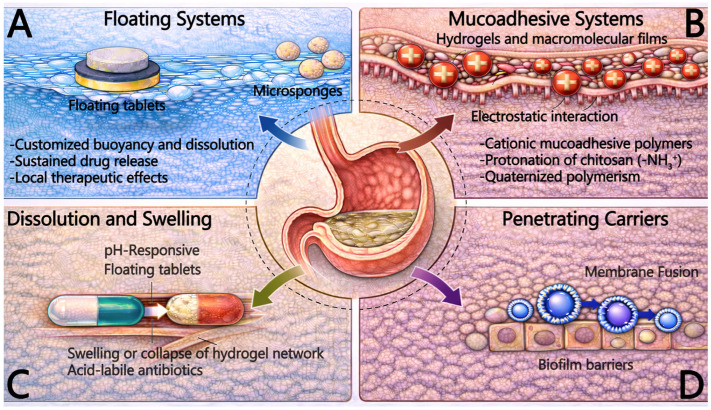
Representative gastric-targeted formulation modalities for prolonged residence and overcoming barriers. This figure summarizes four formulation classes designed to extend gastric residence, increase local drug exposure, and address major intragastric barriers. (**A**) Floating systems. Floating tablets and microsponges use low-density or porous architectures to generate buoyancy and modulate dissolution, enabling sustained drug release and enhanced local efficacy through prolonged gastric retention. (**B**) Mucoadhesive systems. Hydrogels and macromolecular films prolong mucosal contact primarily via electrostatic interactions between cationic mucoadhesive polymers and negatively charged mucins. In acidic environments, chitosan protonation from -NH_2_ to NH_3_^+^-positively strengthens adhesion, whereas quaternized derivatives provide persistent positive charges that support mucoadhesion over a wider pH range and improve resistance to clearance driven by mucus turnover. (**C**) Dissolution and swelling systems. pH-responsive polymer networks undergo swelling or collapse to regulate diffusion and drug release, thereby protecting acid-labile antibiotics and enabling site-adapted delivery. (**D**) Penetrating carriers. Carriers with biofilm-breaching capability promote deeper transport into protected bacterial niches, including designs that facilitate membrane fusion to enhance drug access.

Floating tablets are solid dosage forms engineered to float within the stomach by minimizing their density. By controlling their physical structure, these dosage forms can remain in the stomach for extended periods, preventing premature gastric emptying into the small intestine. Similarly, microsponges and ultra-light porous materials leverage their low-density characteristics to form floating structures within the gastric cavity, retarding drug transit. These materials not only prolong gastric residence time but also facilitate sustained drug release throughout the treatment course, maintaining prolonged local therapeutic effects (Figure 2) [58]. Moreover, such combination treatment strategies have the potential for the eradication of *H. pylori* antibiotic-resistant strains.

The application of 3D printing technology offers enhanced flexibility in the design of gastro-retentive systems. By precisely controlling the pore structure and geometry of printed materials, researchers can optimize both drug release rates and gastric residence time. Compared to traditional formulations, 3D-printed porous tablets allow for the customization of buoyancy and dissolution properties according to specific therapeutic requirements. Advancements in this technology open new avenues for personalized medicine, offering significant advantages, particularly in regimens requiring precise control over drug release kinetics [59,60].

Through these buoyancy-based and retentive systems, drugs can remain in the stomach for extended durations, increasing contact time with the gastric wall and ensuring adequate release at the target site [57]. This is critical for enhancing the local concentration and therapeutic efficacy of antibiotics such as amoxicillin and clarithromycin, especially during and after *H. pylori* eradication therapy, where high local drug concentration is a key determinant for improving eradication rates [58].

### 3.2. Mucoadhesion Strategies: Overcoming the Mucus Turnover Barrier

In gastric-targeted drug delivery systems, utilizing mucoadhesive materials to enhance the affinity between the carrier and the gastric mucosa is a core strategy for prolonging Gastric Residence Time (GRT) and improving local drug bioavailability [59,60]. However, the efficacy of this strategy is significantly limited by the rapid mucus turnover of the gastric mucosa. The gastric mucus layer is a dynamic biological barrier; its continuous process of secretion and degradation results in the constant shedding of the superficial mucus layer, with a turnover cycle typically lasting 4–6 h [61]. This implies that carriers relying solely on non-specific physical adsorption are prone to being cleared along with the shed mucus, leading to “pseudo-adhesion” and drug loss. Consequently, the design of next-generation mucoadhesive delivery systems focuses on establishing stronger intermolecular interactions or achieving deep penetration into the sub-mucous layers to resist the clearance effects induced by mucus turnover (Figure 2) [59,60,61].

To address this physiological barrier, current pharmaceutical strategies primarily focus on optimizing the following mechanisms:

Electrostatic Interaction & Junction Modulation: Chitosan and its derivatives are the most extensively studied cationic mucoadhesive polymers. In the low pH environment of gastric acid, the amino groups on the chitosan chain undergo protonation (-NH_3_^+^), enabling strong electrostatic interactions with the negatively charged sialic acid and sulfonate groups on the side chains of gastric mucin, thereby delaying carrier clearance [62,63,64]. Furthermore, quaternized polymers, by introducing permanent positive charges, further enhance this electrostatic anchoring effect, maintaining adhesion stability across a broader pH range [65]. Notably, chitosan possesses the ability to transiently open intercellular Tight Junctions; this not only enhances paracellular transport but also facilitates the deep penetration of the carrier into the mucosa, thereby circumventing the impact of superficial mucus shedding [66,67].

Covalent Bonding & Thiomers: To overcome the limitations of non-covalent interactions, primarily hydrogen bonds and electrostatic forces, which are susceptible to ionic strength and pH variations, thiolated polymers (Thiomers) have been introduced into the design of gastro-retentive systems. By modifying the polymer backbone with thiol groups (-SH), these polymers can undergo disulfide exchange reactions with cysteine-rich subdomains in mucin, forming stable disulfide bonds [68,69,70]. The strength of this chemical bonding is far superior to physical adsorption and is theoretically capable of significantly prolonging the residence time of the carrier on the gastric mucosal surface, a phenomenon termed mucoadhesion. This potent anchoring mechanism based on covalent bonds effectively overcomes the clearance effects of mucus turnover, providing a chemical basis for long-acting drug delivery [69,70].

Diffusion & Interpenetration Theory: The adhesion mechanism of hydrogels and macromolecular films is primarily based on the Diffusion-Entanglement theory, which posits that when hydrophilic polymers such as Guar gum and Carbomer contact gastric mucus, the polymer chains absorb water, swell, and diffuse into the mucus layer network [71,72]. Due to the movement and mutual interpenetration of molecular chains, physical entanglement occurs between the carrier and mucin chains, accompanied by the formation of numerous hydrogen bonds [72,73]. The efficiency of this mechanism depends on the polymer’s molecular weight, degree of cross-linking, and hydration rate. Optimized hydrogel systems can form a viscoelastic gel layer instantly upon contact with the gastric wall; this layer not only resists the shear forces of gastric peristalsis but also serves as a drug depot, providing a continuous concentration gradient on the mucosal surface to drive passive drug diffusion [71,72,73].

### 3.3. Microenvironment Responsiveness and Multimodal Release: Intelligent Delivery Based on Pathological Features

Stimuli-responsive drug delivery systems (DDS) aim to utilize the physiological environment of the stomach or the specific pathological microenvironment of *H. pylori* infection foci to achieve spatiotemporal controlled release. Compared to passive diffusion, this on-demand release strategy elevates drug concentration at the lesion site while reducing toxic side effects on non-targeted tissues [74,75,76]. Current strategies primarily focus on three major endogenous triggers: pH gradients, specific enzymes, and reactive oxygen species (ROS), as well as exogenous triggers such as photothermal and photodynamic therapies [75,76].

#### 3.3.1. pH-Responsive: Utilizing Acidic Gradients for Dissolution and Swelling

Given the significant disparity between the highly acidic gastric lumen (pH 1.0–3.0) and the near-neutral environment of the sub-mucous layer, pH responsiveness represents the most classic gastric-targeting strategy [77,78,79]. These systems are typically based on polymers containing ionizable groups like carboxyl or amino moieties, such as polymethacrylic acid and the Eudragit^®^ series [77,78]. Mechanistically, protonation or deprotonation induces electrostatic repulsion within the polymer chains, leading to the swelling or collapse of the hydrogel network, thereby controlling drug diffusion [78,79]. Furthermore, for acid-labile antibiotics, exemplified by clarithromycin, or proton pump inhibitors (PPIs), pH-responsive carriers can be designed to remain intact within gastric acid to protect the payload, while triggering release only upon contact with the higher pH environment of the mucus layer or epithelial interface, achieving a dual function of protection and targeting (Figure 2) [77,78,79].

#### 3.3.2. Enzyme-Responsive: Targeting the Signature Enzyme of *H. pylori*

To survive in gastric acid, *H. pylori* secretes high concentrations of urease to decompose urea into ammonia. This specific biomarker is utilized to design highly selective drug release systems [80,81]. For instance, by incorporating urea components or pH-sensitive gating molecules into the carrier, the local microenvironmental pH rises sharply due to ammonia production upon contact with bacterial urease; alternatively, the carrier backbone may be directly hydrolyzed by the enzyme, triggering a burst release of the drug [82]. This biomarker-triggered strategy not only enhances targeting precision towards infection foci but also effectively minimizes drug leakage in non-infected regions, such as the intestine [82,83].

#### 3.3.3. ROS-Responsive: Dual-Function Anti-Inflammation and Release

**Figure 3:**
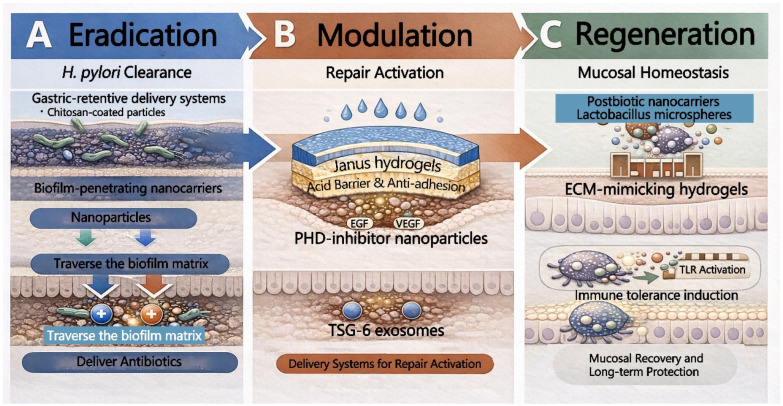
Pharmaceutical strategies aligned with the Eradication–Modulation–Regeneration framework for gastric remodeling after *H. pylori* therapy. (**A**) Eradication. Gastro-retentive delivery systems such as chitosan-coated carriers and biofilm-penetrating nanocarriers are designed to traverse the biofilm matrix and increase local antibiotic exposure at the colonization niche, thereby improving bacterial clearance. (**B**) Modulation. Spatiotemporally controllable biomaterials such as Janus hydrogels provide an acid barrier and anti-adhesion protection while functioning as depots for pro-repair signals, including VEGF. In parallel, immunomodulatory platforms, represented by nanoparticles delivering prolyl hydroxylase inhibitors and TSG-6–enriched exosomes, are used to dampen excessive inflammation and establish a permissive microenvironment for tissue repair. (**C**) Regeneration. Microecology-oriented systems, including postbiotic-loaded nanocarriers, Lactobacillus-based microspheres, and extracellular matrix mimetic hydrogels, promote immune tolerance through pattern-recognition signaling such as TLR pathways and reinforce mucosal recovery and sustained protection, ultimately restoring durable mucosal homeostasis.

*H. pylori* infection induces neutrophil infiltration and the production of massive amounts of reactive oxygen species (ROS), resulting in local oxidative stress levels significantly higher than those in normal tissue [84,85]. This provides an ideal pathological trigger point for designing ROS-responsive carriers. By constructing carriers using oxidation-sensitive chemical bonds, including thioketal, phenylboronic ester, or diselenide linkages, allowing the carrier to disintegrate and release the drug upon reaching the high-ROS inflammatory microenvironment [86]. More importantly, this process consumes excess ROS, permitting the carrier itself to function as an ROS scavenger. This dual mechanism of drug release, accompanied by antioxidation, not only eradicates the pathogen but also helps block the oxidative stress-mediated inflammatory vicious cycle, accelerating gastric mucosal repair (Figure 3) [87].

#### 3.3.4. Light-Triggered and Multimodal Synergy

Beyond endogenous stimuli, light-triggered systems utilize photosensitive materials to generate thermal energy (Photothermal Therapy, PTT) or singlet oxygen (Photodynamic Therapy, PDT). These approaches not only physically disrupt bacterial structures but also accelerate drug release through thermal effects [88,89]. To address recalcitrant biofilms and drug resistance, modern delivery systems are evolving toward multimodal synergy. For example, carriers integrating nanozymes or metal ions possess enzyme-responsive release capabilities while simultaneously simulating peroxidase or superoxide dismutase (SOD) activity to perform in situ catalysis—generating bactericidal ROS or scavenging inflammatory ROS. This combinatorial cocktail strategy, which integrates anti-biofilm activity, immunomodulation, and precision delivery, represents the future direction of gastric-targeted drug administration [86,88,89]. Representative gastric-targeted delivery systems discussed in this section are summarized in Table 1.

## 4. The Role of Gastric-Targeted Delivery in the “Eradication–Modulation–Regeneration” Pathway

### 4.1. The Eradication Phase: Breaching Biofilm Barriers and Precise Eradication

In the eradication phase (Figure 3A) of gastric-targeted delivery, the primary therapeutic objective is to surmount the unique physiological and pathological barriers of the stomach to achieve effective accumulation and penetration of bactericidal agents at the infection site. Conventional oral antibiotic therapies are often compromised by rapid gastric emptying. This rapid transit causes local drug concentrations to fall below the minimum inhibitory concentration (MIC). Furthermore, these therapies face significant challenges in penetrating the dense biofilm constructed by *H. pylori* [105,106]. Consequently, the development of intelligent delivery systems that integrate gastric retention with efficient biofilm penetration has become pivotal for improving eradication rates [107].

To address the physiological challenge of rapid gastric emptying, gastro-retentive drug delivery systems (GRDDS) and floating formulations serve as an effective first line of defense. Utilizing low-density floating or mucoadhesion mechanisms, these carriers significantly prolong the gastric residence window, ensuring sustained high-concentration exposure of antibiotics on the mucosal surface [108]. However, for refractory infections, merely enhancing surface concentration is often insufficient for *H. pylori* eradication. *H. pylori* tends to form biofilms composed of extracellular polymeric substances (EPS) deep within gastric pits. These biofilms act as sturdy physical barriers that drastically impede the penetration of conventional small-molecule antibiotics, thereby leading to the pseudo-resistance clinical phenomenon [109,110].

To surmount this pathological barrier, nano-targeted delivery systems exhibit unique advantages over traditional formulations. Leveraging their minute size effect, nanocarriers can penetrate the mucus layer and infiltrate the biofilm matrix. Advanced designs endow carriers with the capability to disrupt EPS; for instance, utilizing chitosan or functionalized metal nanozymes to directly degrade the biofilm structure exposes concealed bacteria to antibiotic attack [111]. Furthermore, intelligent charge-reversal strategies are widely applied to enhance penetration efficiency: carriers maintain a negative or neutral charge while traversing the mucus layer to minimize resistance, but rapidly revert to a positive charge upon contact with the more acidic biofilm microenvironment or bacterial surface [112]. This dynamic modulation of surface charge not only promotes electrostatic adsorption between the carrier and the bacteria but also exerts synergistic bactericidal effects by disrupting bacterial membrane potential (Figure 2) [112].

Building on these foundations, multi-mechanism synergy strategies further elevate eradication efficiency. Modern delivery systems are no longer confined to single-drug transport but tend to construct multifunctional combinatorial platforms. For instance, designs such as amoxicillin-alginate/chitosan complexes or clarithromycin-pantoprazole bilayer tablets can synchronously release proton pump inhibitors (PPIs) or urease inhibitors alongside antibiotics [113]. The former reduces antibiotic degradation by suppressing gastric acid secretion, whereas the latter directly disrupts the protective ammonia-rich microenvironment that supports bacterial survival. Collectively, this integrated strategy combining gastric retention, biofilm penetration, and microenvironment modulation shifts conventional therapy from passive exposure to active site-specific intervention, thereby strengthening the eradication of resistant strains and biofilm-associated *H. pylori*.

### 4.2. The Modulation and Regeneration Phase: Spatiotemporally Controlled Regeneration and Microenvironment Remodeling

In the regeneration phase of gastric-targeted delivery, the therapeutic focus extends beyond pathogen eradication to tissue regeneration and microenvironment remodeling (Figure 3C). Effective platforms are expected to deliver bioactive cues with spatiotemporal control, ensuring stable lesion coverage for acid protection while matching the dynamics of epithelial repair and angiogenesis. In this context, functionalized hydrogels, engineered exosomes, and smart nanocarriers have been extensively explored to promote ulcer healing, vascularization, and redox homeostasis. [114,115,116].

First, regarding the supply of biological signals required for repair, hydrogel systems loaded with growth factors and bioactive molecules play a pivotal role [117,118]. Macromolecules such as EGF, VEGF, and TFFs are highly susceptible to deactivation in gastric acid; thus, carriers must act as a protective barrier and a sustained-release depot. By encapsulating these factors within a biocompatible matrix, delivery systems can mimic the body’s secretion patterns, continuously releasing signaling molecules to stimulate re-epithelialization and angiogenesis in the damaged region [118]. Concurrently, the introduction of natural active ingredients such as butyrate and resveratrol specifically targets the high levels of oxidative stress remaining from *H. pylori* infection, creating a low-toxicity metabolic environment for nascent cells by scavenging ROS, thereby accelerating the metabolic process of tissue repair [119].

In terms of spatial structural innovation, Janus hydrogels represent a prototypical platform for site-specific mucosal repair. In contrast to conventional homogeneous hydrogels, Janus hydrogels feature an asymmetric, dual-faced architecture that enables the concurrent achievement of hemostatic sealing and anti-adhesive protection. The tissue-contacting side is typically functionalized with tissue-affinity moieties to ensure firm adhesion to the ulcer bed, thereby enabling rapid hemostasis and providing an interface for localized drug release. Conversely, the backing side is designed as a smooth, hydrophobic layer resistant to mucoadhesion, effectively blocking gastric acid erosion and preventing pathological adhesion with surrounding tissues [120]. This dual functionality of treatment and protection spatial segmentation, combined with low-swelling characteristics, not only avoids mechanical detachment caused by excessive water absorption but also provides a stable physical barrier for the fragile nascent mucosa, ensuring the repair process proceeds without external interference [121].

Furthermore, the modulation of the immune microenvironment acts as a bridge connecting eradication and regeneration (Figure 3B). To induce the transition of the immune response from a pro-inflammatory to an anti-inflammatory state, immunomodulatory carriers, notably tumor necrosis factor-stimulated gene-6 (TSG-6) exosomes and prolyl hydroxylase domain (PHD) inhibitor nanoparticles, have been developed for precise intervention in local inflammatory signals. For instance, TSG-6 exosomes minimize local cytokine storms by inhibiting the release of pro-inflammatory cytokines; meanwhile, PHD inhibitors regulate the Hypoxia-Inducible Factor (HIF) pathway, enhancing the adaptability of the gastric mucosa to hypoxic environments at the cellular and molecular levels, while promoting anti-inflammatory responses [122,123]. AI-assisted design of small-molecule drugs further improves the precision of this modulation, enabling delivery systems to function like an immune modulator, maintaining the local microenvironment within the optimal homeostatic window for tissue regeneration [124].

### 4.3. Homeostasis Reconstruction: Microbiota Remodeling and Long-Term Barrier Maintenance

Following pathogen eradication and damage repair, the ultimate therapeutic goal shifts to Homeostasis Reconstruction. This phase aims to break the vicious cycle of infection-inflammation-recurrence by restoring long-term immune tolerance and microecological balance in the gastric mucosa [125,126]. To this end, the biomaterial-mediated Eradicate–Modulate–Regenerate (EMR) strategy has been proposed as a core framework for homeostasis maintenance. This strategy emphasizes eradicating residual pathogenic threats, modulating the local immune microenvironment, and finally solidifying therapeutic outcomes through sustained barrier regeneration. Under this framework, drug delivery systems based on microecological modulation have become key tools for maintaining long-term homeostasis [127].

In microbiota remodeling strategies, engineered probiotics and *Lactobacillus* microspheres have demonstrated significant potential [128,129,130]. These active preparations inhibit pathogen recolonization via competitive exclusion mechanisms and secrete metabolites such as SCFAs to enhance mucin expression. However, live probiotic preparations face clinical challenges, including low survival rates in gastric acid, high uncertainty in colonization, and the risk of inducing bacteremia in immunocompromised patients [131,132,133].

Addressing these limitations, Postbiotics—defined as inanimate bacteria and their metabolic products or cellular components, such as teichoic acids and peptidoglycans—have emerged as a superior novel therapeutic agent [134,135]. Compared to live probiotics, postbiotics possess distinct pharmaceutical advantages, primarily characterized by superior physicochemical stability and enhanced safety [136]. Specifically, they do not require cold-chain transport and are impervious to destruction by gastric acid or bile salts, thereby ensuring delivery to the target site at defined dosages. Furthermore, they demonstrate lower immunogenicity and circumvent the risk of live bacterial translocation infection, making them particularly suitable for gastric patients with compromised barrier functions [137,138]. Mechanistically, research indicates that postbiotics encapsulated in nanocarriers can similarly activate host Toll-like receptors (TLRs) to induce anti-inflammatory cytokine secretion and strengthen epithelial tight junctions, thereby efficiently achieving dual homeostatic regulation of microecology and immunity in the absence of live bacterial colonization [139].

To further consolidate homeostasis, the design of biomaterials is evolving from simple drug carriers to “Biomimetic Niches.” Utilizing hydrogels or porous scaffolds to mimic the natural Extracellular Matrix (ECM) of the gastric mucosa provides physical support for the colonization of endogenous beneficial microbiota and the differentiation of stem cells [140]. These materials continuously release anti-inflammatory molecules to maintain a pro-resolution microenvironment. Simultaneously, they physically shield the nascent epithelium from gastric acid irritation, thereby preventing the resurgence of inflammation. This comprehensive EMR system, integrating antibacterial, anti-inflammatory, and barrier-support functions, provides a long-acting protective mechanism to prevent the progression of gastritis toward atrophy and intestinal metaplasia [141,142]. These delivery systems transform the biological understanding of immune modulation into actionable therapeutic strategies by ensuring precise spatiotemporal drug release.

## 5. Existing Gastric-Targeted Delivery Formulations and Clinical Translation

### 5.1. Antibiotic Gastro-Retentive Formulations: From Extended Retention to Enhanced Efficacy and Reduced Toxicity

Traditional oral antibiotics often suffer from insufficient local concentrations due to rapid gastric emptying, whereas novel gastro-retentive formulations markedly improve the eradication efficiency of *H. pylori* by optimizing pharmacokinetics [90,91,143]. Research indicates that amoxicillin-alginate/chitosan nanoparticles, utilizing electrostatic adsorption mechanisms, not only prolong the therapeutic window of antibiotics on the gastric mucosal surface but also substantially decrease direct toxicity to epithelial cells, achieving a balance between potent bactericidal activity and mucosal protection [90,91]. In terms of solid dosage forms, low-density floating tablets and microsponges have been confirmed to effectively resist gastric emptying; by maintaining a high steady-state concentration of amoxicillin within the stomach, they improve the efficacy of eradication therapy and reduce systemic side effects. Furthermore, the application of 3D printing technology has further realized personalized drug delivery. By precisely regulating the porosity and mechanical strength of porous tablets, researchers have successfully customized drug release profiles to match the specific gastric motility characteristics of patients, providing a new paradigm for the precision treatment of refractory infections [90,91,92].

### 5.2. Nanocarriers and Composite Carriers: Multifunctional Synergy and Precision Delivery

Nanocomposite carriers have moved beyond conventional drug-transport roles and are increasingly being developed as integrated platforms that combine therapeutic and diagnostic functionalities, as well as multimodal therapeutic synergy [93]. Mesoporous Silica Nanoparticles (MSNs) and Metal–Organic Frameworks (MOFs), distinguished by their high specific surface areas and tunable pore sizes, are extensively employed to address the loading challenges of poorly soluble drugs. Studies indicate that MSNs can achieve a drug loading capacity of up to 300 mg/g for clarithromycin, with an encapsulation efficiency exceeding 85%, significantly surpassing conventional formulations [94].

To breach the bottleneck of antibiotic resistance, functional modification has emerged as a research hotspot. For instance, quaternized chitosan-β-cyclodextrin nanoparticles significantly enhance antibiotic bioavailability by intensifying electrostatic interactions with bacterial surfaces [95]. Concurrently, MSNs-resveratrol complexes integrate sustained-release technology with the antioxidant activity of natural products, effectively alleviating infection-induced oxidative stress injury while exerting bactericidal effects [96]. Even more innovative is the MOF-metal nanozyme composite system, which integrates in situ catalytic capabilities to generate ROS that directly disrupt bacterial biofilms. This antibiotic–nanozyme combination strategy has been associated with improved eradication of biofilm-associated *H. pylori* compared with monotherapy in preclinical studies. [97].

### 5.3. Functional Hydrogels and Films: Physical Barriers and Regenerative Microenvironments

In the field of gastric mucosal repair, the application of functional hydrogels and films focuses on constructing physical barriers conducive to ulcer healing [144]. The asymmetric design of Janus hydrogels addresses a dual clinical necessity: their adhesive side provides immediate hemostasis and local drug delivery capabilities, whereas the backing side effectively blocks gastric acid erosion and prevents tissue adhesion [98]. This structural advantage has shown excellent performance in accelerating ulcer healing.

In addressing the high-shear environment within the stomach, low-swelling adhesive hydrogels prevent structural disintegration caused by excessive swelling through optimized cross-linking networks, ensuring the sustained release of anti-inflammatory drugs [99]. Furthermore, marine-inspired mussel-biomimetic films utilize catechol groups to achieve potent tissue anchoring in wet environments [100]. Research confirms that these biomimetic films can adhere tightly to the ulcer surface, functioning as a bio-mimetic wound dressing, isolating gastric acid irritation while slowly releasing repair factors, thereby significantly shortening the epithelial regeneration cycle [101].

### 5.4. Probiotics: Microecological Regulation and Immune Remodeling

Probiotics and biogenic preparations have emerged as critical adjunctive modalities for gastric mucosal repair due to their intrinsic immunomodulatory activities [102,145]. Addressing the challenge of low survival rates of live bacteria in gastric acid, *Lactobacillus* microspheres and engineered probiotics have demonstrated significantly improved gastric colonization capabilities through physical encapsulation or genetic modification. These strategies leverage mechanisms of competitive exclusion to inhibit pathogens while secreting SCFAs to nourish the epithelium [102].

Furthermore, to circumvent the risks associated with live bacterial infection, exosomes, Bacillus Calmette-Guérin (BCG), and relevant derivatives have garnered significant attention as cell-free therapeutic strategies [103]. Leveraging their nanoscale size and natural homologous targeting properties, exosomes can efficiently penetrate the mucus layer to deliver miRNAs or anti-inflammatory proteins, directly modulating macrophage polarization. Meanwhile, BCG microparticles enhance the host capacity to clear residual pathogens by activating local innate immunity. Collectively, these approaches, which include bacterial antagonism-based interventions and cell-free immunotherapeutic modalities, may offer biocompatible and sustained options for managing antibiotic-resistant infections [104].

### 5.5. Natural Products and Traditional Medicines: Molecular Mechanism-Driven Synergistic Repair

Despite the multi-target pharmacological activities possessed by natural products, their clinical translation is often restricted by low water solubility and significant first-pass effects [146]. Gastric-targeted delivery systems significantly potentiate the intervention efficacy of these phytochemical constituents at the molecular level through mechanisms of physical retention and nano-solubilization [147].

Regarding inflammation regulation, various phytochemical molecules exhibit inhibitory effects on core inflammatory pathways [148]. Aloe emodin and curcumin have been confirmed to suppress nuclear factor kappa B (NF-κB) and Toll-like receptor 4 (TLR4) signaling cascades, directly downregulating the expression of pro-inflammatory cytokines such as TNF-α; conversely, terpenoids derived from gardenia oil primarily target the inhibition of cyclooxygenase-2 (COX-2) enzyme activity, thereby mitigating inflammatory responses while maintaining mucosal blood perfusion [149,150].

In the context of antioxidation and barrier remodeling, curcumin acts as a potent activator of the Nrf2/HO-1 pathway (nuclear factor erythroid 2-related factor 2, heme oxygenase-1), inducing endogenous antioxidant enzymes to scavenge excess ROS generated by infection [151]. Simultaneously, aloe polysaccharides and flavonoids (such as quercetin) accelerate Extracellular Matrix (ECM) remodeling and the closure of physical barriers by promoting Fibroblast Growth Factor (FGF)secretion and upregulating the expression of tight Junction proteins, such as Claudin and Occludin, respectively [152].

To surmount these physicochemical limitations, floating tablets and Nanostructured Lipid Carriers (NLCs) have been extensively employed [153]. These formulations not only prolong gastric residence and local exposure of bioactive molecules but also markedly improve the apparent solubility of hydrophobic constituents, thereby enabling integrated anti-inflammatory, antioxidant, and mucosal-repair effects [154,155].

### 5.6. Clinical Translation and Real-World Evidence: From Evidence-Based Medicine to Precision Medicine

Although novel gastric-targeted delivery systems have shown promising efficacy in preclinical studies, successful translation ultimately depends on demonstrating the clinical relevance of achieving sustained high local drug exposure and restoring microecological homeostasis [156,157]. In recent years, medical-evidence based on High-Dose Dual Therapy (HDDT) and microbiota-combined therapies has indirectly supported this pharmaceutical strategy [158].

HDDT approximates the prolonged high local antibiotic exposure achieved by gastro-retentive formulations by employing high-frequency dosing schedules [159]. Meta-analyses indicate that compared to traditional quadruple therapy, HDDT achieves comparable eradication rates (85.5% vs. 87.2%) but with substantially decreased adverse events (14.4% vs. 40.4%, risk ratio = 0.42) [160]. Notably, Vonoprazan-based HDDT regimens can achieve eradication rates as high as 91.3% in refractory populations. These data provide reverse validation for the core pharmaceutical hypothesis: efficient eradication can be achieved without complex multi-drug combinations, provided that sufficient local concentration and an appropriate pH environment are maintained. Furthermore, probiotic-combined therapy has been confirmed to increase eradication rates by approximately 10% while reducing side effects by 50%, highlighting the critical role of microecological homeostasis in adjuvant therapy [161].

However, Real-World Evidence (RWE) reveals the limitations of existing therapies, which are significantly compromised by patient compliance. This presents precisely the entry point for Gastro-Retentive Drug Delivery Systems (GRDDS): transforming multi-dose regimens into once-daily smart retentive formulations [162]. By eliminating the compliance variable at the formulation level, GRDDS bridges the gap between Randomized Controlled Trials (RCTs) and real-world outcomes. Future translational research should prioritize the dosage-form development of HDDT regimens and evaluate their long-term effectiveness and safety in multicenter clinical studies [163].

## 6. Conclusions and Future Perspectives

As a precision-oriented therapeutic approach, gastric-targeted drug delivery shows considerable potential to enhance efficacy, reduce off-target toxicity, and promote mucosal healing across the continuum from *H. pylori* eradication to gastric mucosal repair. By optimizing drug delivery systems, it is possible to elevate local drug concentrations within the stomach, prolong gastric residence time, and minimize mucosal injury. Nevertheless, gastric-targeted delivery still faces multiple challenges, necessitating further optimization and exploration in areas such as personalized medicine, combination therapies, translational applications, real-world validation, and the development of advanced models [164,165].

### 6.1. Personalization

Individual variations in gastric motility, gastric acid secretion, gut microbiota, and antibiotic resistance profiles present challenges for the clinical application of gastric-targeted delivery [166]. In the realm of personalized medicine, formulating treatment regimens tailored to patient-specific physiological characteristics and disease states has become a focal point of current research. Differences in gastric motility among patients can lead to variations in the gastric residence time of drugs, thereby influencing therapeutic efficacy [167]. Furthermore, discrepancies in acid secretion, the composition of the gastrointestinal microbiota, and *H. pylori* resistance profiles may result in divergent outcomes for the same treatment regimen across different patients. Therefore, establishing stratification strategies based on biomarkers and companion diagnostic systems is key to optimizing treatment outcomes [168]. By precisely identifying patients’ physiological traits and microecological states, it is possible to realize more personalized treatment regimens, enhancing efficacy while reducing adverse reactions.

### 6.2. Combination Therapies

Gastric-targeted delivery strategies are increasingly adopting multimodal formulations. By integrating antimicrobial, anti-inflammatory, reparative, and probiotic agents, these systems significantly enhance therapeutic efficacy. Moreover, they improve the quality of homeostasis reconstruction [169,170]. Multimodal therapy, by utilizing the combined application of drugs with distinct mechanisms of action, can simultaneously inhibit pathogens, alleviate inflammation, and facilitate gastric mucosal repair. For instance, antibiotics serve to eradicate *H. pylori*, anti-inflammatory agents mitigate gastric mucosal inflammatory responses, repair factors promote cellular regeneration, while probiotics modulate the intestinal microecology to restore gastrointestinal barrier function. However, the clinical application of these combination therapies still faces challenges regarding drug–drug interactions and therapeutic timing [171]. Optimizing the dosing sequence, duration, and dosage of these agents remains a critical issue that necessitates resolution. By integrating preclinical studies with randomized controlled trials (RCTs), the optimization of treatment protocols is poised to further elevate the cure rates of gastric diseases and mitigate the risk of patient resistance [172].

### 6.3. Translational Barriers, Manufacturing Processes, and Pharmacoeconomic Considerations

Although gastric-targeted drug delivery systems have generated compelling results at the laboratory stage, their clinical translation remains constrained by three major barriers: Chemistry, Manufacturing, and Controls (CMC) readiness, regulatory evaluation, and pharmacoeconomic feasibility [173].

First, batch-to-batch consistency in large-scale manufacturing serves as the primary bottleneck hindering the market launch of novel formulations. The preparation processes for nanomedicines, smart hydrogels, and biogenic preparations, such as exosomes, are typically highly complex, involving intricate chemical modifications or biological fermentation processes. When scaling up from milligram-level laboratory preparation to kilogram-level industrial production, a scale-up effect often occurs, leading to deviations in particle size distribution, drug encapsulation efficiency, or release kinetics. Therefore, the concept of Quality by Design (QbD) must be introduced to establish a rigorous control system for Critical Quality Attributes (CQAs), ensuring high uniformity in physicochemical properties and biological activity for every batch of carriers [174].

Second, the lag in regulatory pathways adds uncertainty to the approval process. Current pharmaceutical regulatory frameworks primarily target traditional small-molecule drugs or single biologics. In contrast, novel gastric-targeted systems often fall under the more complex categories of drug–device combinations or nanomedicines [175]. For instance, multimodal carriers often integrate diagnostic, therapeutic, and microenvironment-modulating functions. Currently, however, there is a lack of internationally harmonized guidelines to define their primary mode of action. Furthermore, standardized protocols for assessing their long-term in vivo biodegradability and toxicity remain to be established [176,177]. This necessitates early communication between researchers and regulatory agencies to drive the establishment of standardized assessment systems tailored for novel delivery systems.

Finally, pharmacoeconomic feasibility is a key determinant of whether novel formulations can be adopted at scale in routine clinical practice. Because *H. pylori* infection is highly prevalent worldwide, standard antibiotic-based regimens remain inexpensive and widely accessible. Consequently, if manufacturing and distribution costs for advanced gastric-targeted formulations are substantially higher, their positioning as first-line options will be constrained by affordability and reimbursement considerations. Therefore, formulation development should balance technological sophistication with cost containment to ensure patient access while preserving clinical value [178]. Future research and development should prioritize streamlined, scalable manufacturing workflows and the use of cost-effective pharmaceutical excipients. In parallel, the intended clinical use case for these advanced formulations should be explicitly defined. For instance, they may be preferentially positioned for refractory infection or multidrug-resistant *H. pylori* in which conventional regimens have failed [179]. By significantly reducing the costs associated with complications arising from treatment failure (such as gastric cancer), these advanced systems can demonstrate their distinct pharmacoeconomic advantages.

### 6.4. Advanced Models: From Static In Vitro Systems to Gastric Organoid-on-a-Chip Platforms

To bridge the translational gap between preclinical research and clinical translation, constructing high-fidelity in vitro evaluation models has become increasingly paramount. Traditional two-dimensional (2D) cell lines lack spatial structure, while animal models exhibit significant species inter-variability [180]. Against this backdrop, the Gastric Organoid-on-a-Chip, a revolutionary platform fusing stem cell biology with microfluidic technology, is emerging as a critical translational bridge connecting in vitro experiments with in vivo trials.

The Gastric Organoid-on-a-Chip represents an emerging preclinical tool that extends beyond the simple culture of organoids. By simulating physiological fluid shear stress and periodic mechanical stretching, it offers a more physiologically relevant model for screening formulations, although its standardization for high-throughput analysis remains to be established. This dynamic physical stimulation is crucial for maintaining gastric epithelial polarity and promoting the continuous secretion and renewal of functional mucus, thereby perfectly reproducing the authentic mucus turnover barrier in vitro [181]. This capability allows researchers, for the first time, to precisely evaluate the true retention capacity of mucoadhesive materials against mucus clearance and the penetration kinetics of nanocarriers under flow conditions within a highly biomimetic dynamic environment.

More uniquely, the Organoid-on-a-chip provides a visualization window for studying complex host-microbe interactions. The system allows for the precise inoculation of *H. pylori* into the luminal side of the chip, recreating bacterial colonization within the dynamic mucus layer, biofilm formation, and the trans-endothelial recruitment of immune cells [182]. This humanized pathological model enables deep mechanistic analysis of cellular-level responses throughout the entire Eradication–Modulation–Regeneration process. When combined with AI-driven high-throughput image analysis and predictive algorithms, this platform promises to drastically improve the accuracy of candidate formulation screening, shorten new drug research and development cycles, and significantly reduce the risk of clinical trial failure.

## 7. Epilogue

The therapeutic paradigm for *H. pylori* is undergoing a profound transformation from singular *H. pylori* eradication to holistic gastric health reconstruction. Future gastric-targeted drug delivery systems will no longer function merely as vehicles for antibiotics, but as intelligent platforms integrating biofilm penetration, immune microenvironment remodeling, and mucosal barrier repair.

With the cross-disciplinary convergence of materials science, immunology, and organoid technology, we have every reason to believe that this integrated Eradication–Modulation–Regeneration strategy will provide the ultimate solution for thoroughly resolving *H. pylori* infection and its associated gastric diseases. This approach aims to shift the therapeutic paradigm from mere pathogen eradication to the holistic restoration of gastric mucosal homeostasis.

## Figures and Tables

**Table 1:** Summary of Representative Gastric-Targeted Delivery Systems.

Formulation Modalities	Typical Payloads	Key Design Features/Mechanism	Advantages	Representative Refs.
Amoxicillin alginate chitosan nanoparticles	Amoxicillin	Electrostatic adsorption to mucosa; improves local exposure while limiting direct cytotoxicity	Extended therapeutic window; improved efficacy with lower off-target toxicity	[90,91]
Low-density floating tablets; microsponges	Amoxicillin (typical)	Buoyancy-driven retention; sustained release	Higher steady-state gastric levels; reduced systemic side effects	[90,91,92]
3D-printed porous gastroretentive systems	Antibiotics (typical)	Porosity and mechanical strength tuning via 3D printing	Patient-tailored release; potential for refractory infections	[90,91,92]
MSNs; MOFs for poorly soluble drugs	Curcumin; clarithromycin	High surface area and tunable pores; pH-responsive release behavior	Better solubility and controlled release in gastric milieu	[93,94]
Quaternized chitosan β-cyclodextrin nanoparticles	Antibiotics (typical)	Increased electrostatic interaction with bacterial surfaces	Improved antibiotic bioavailability and antibacterial efficacy	[95]
MSNs resveratrol complexes	Resveratrol (plus antibiotics in some designs)	Sustained release plus intrinsic antioxidant activity	Alleviates ROS-associated injury while supporting bactericidal effects	[96]
MOF metal nanozyme composite	Antibiotics and catalytic component	In situ catalytic ROS generation to damage biofilm matrix; synergistic killing	Improved eradication vs. monotherapy in biofilm-associated *H. pylori*	[97]
Asymmetric Janus hydrogel	Local drugs; hemostatic/repair cues	Adhesive tissue-facing layer for hemostasis and delivery; backing layer blocks acid erosion and reduces adhesion	Accelerated ulcer healing with concurrent protection	[98]
Low-swelling adhesive hydrogels	Anti-inflammatory drugs	Optimized cross-linking network limits over-swelling and detachment	Sustained release with improved mechanical stability	[99]
Mussel-biomimetic catechol-based films	Repair factors (typical)	Catechol-mediated tissue anchoring; barrier plus slow release	Shortens epithelial regeneration cycle; protects nascent mucosa	[100,101]
Lactobacillus microspheres	Live bacteria; metabolites	Encapsulation or genetic modification improves acid survival; competitive exclusion; SCFA secretion	Improved colonization; supports epithelial nutrition and barrier function	[102]
Exosomes delivering miRNAs/proteins; BCG microparticles	miRNAs; anti-inflammatory proteins; immunostimulatory components	Mucus penetration and homologous targeting for exosomes; innate immune activation for BCG derivatives	Biocompatible and sustained options for antibiotic-resistant contexts	[103,104]

## Data Availability

No new data were created for this study. All data analyzed in this review are available in the publications cited in the references section.

## Abbreviations

The following abbreviations are used in this manuscript:

AI	artificial intelligence
BCG	Bacillus Calmette–Guérin
CagA	cytotoxin-associated gene A
CMC	Chemistry Manufacturing and Controls
COX-2	cyclooxygenase-2
CQAs	critical quality attributes
DDS	drug delivery systems
ECM	extracellular matrix
EGF	epidermal growth factor
EGFR	epidermal growth factor receptor
EMR	Eradicate-Modulate-Regenerate
EPS	extracellular polymeric substances
GRDDS	gastro-retentive drug delivery systems
GRT	gastric residence time
HDDT	high-dose dual therapy
HIF	hypoxia-inducible factor
HO-1	heme oxygenase-1
IFN-γ	interferon-γ
IL-10	interleukin-10
MHC	major histocompatibility complex
MIC	minimum inhibitory concentration
miRNAs	microRNAs
MOFs	metal–organic frameworks
MSNs	mesoporous silica nanoparticles
NF-κB	nuclear factor kappa B
NLCs	nanostructured lipid carriers
Nrf2	nuclear factor erythroid 2-related factor 2
PHD	prolyl hydroxylase domain
PDT	photodynamic therapy
PPIs	proton pump inhibitors
PTT	photothermal therapy
QbD	quality by design
RCTs	randomized controlled trials
ROS	reactive oxygen species
RNS	reactive nitrogen species
RR	risk ratio
RWE	real-world evidence
SCFAs	short-chain fatty acids
SOD	superoxide dismutase
TFFs	trefoil factor family
TFF2	trefoil factor 2
TGF-β	transforming growth factor-β
TJs	tight junctions
TLRs	toll-like receptors
TLR4	Toll-like receptor 4
TNF-α	tumor necrosis factor-α
Tregs	regulatory T cells
Th1	T helper 1
Th17	T helper 17
TSG-6	tumor necrosis factor-stimulated gene-6
VacA	vacuolating cytotoxin A.
